# Optimizing computational methods of modeling vertebroplasty in experimentally augmented human lumbar vertebrae

**DOI:** 10.1002/jsp2.1077

**Published:** 2020-02-03

**Authors:** Gavin A. Day, Alison C. Jones, Ruth K. Wilcox

**Affiliations:** ^1^ Institute of Medical and Biological Engineering, Mechanical Engineering University of Leeds Leeds UK

**Keywords:** Scement augmentation, finite element, model, vertebra, vertebroplasty, vertebral fracture

## Abstract

Vertebroplasty has been widely used for the treatment of osteoporotic compression fractures but the efficacy of the technique has been questioned by the outcomes of randomized clinical trials. Finite‐element (FE) models allow an investigation into the structural and geometric variation that affect the response to augmentation. However, current specimen‐specific FE models are limited due to their poor reproduction of cement augmentation behavior. The aims of this study were to develop new methods of modeling the vertebral body in both a nonaugmented and augmented state. Experimental tests were conducted using human lumbar spine vertebral specimens. These tests included micro‐computed tomography imaging, mechanical testing, augmentation with cement, reimaging, and retesting. Specimen‐specific FE models of the vertebrae were made comparing different approaches to capturing the bone material properties and to modeling the cement augmentation region. These methods significantly improved the modeling accuracy of nonaugmented vertebrae. Methods that used the registration of multiple images (pre‐ and post‐augmentation) of a vertebra achieved good agreement between augmented models and their experimental counterparts in terms of predictions of stiffness. Such models allow for further investigation into how vertebral variation influences the mechanical outcomes of vertebroplasty.

## Graphical abstract

Vertebroplasty has been widely used for the treatment of osteoporotic compression fractures but the efficacy of the technique has been questioned by the outcomes of randomized clinical trials. Current specimen‐specific finite‐element models are limited due to their poor reproduction of cement augmentation behavior and so struggle to identify the mechanical response to vertebroplasty. Through validation against experimentally tested and augmented vertebrae, the methods developed in this study allow for an improvement in the modeling accuracy of augmented vertebral specimens.

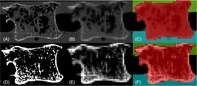

## 1 INTRODUCTION

Vertebral compression fractures are among the most common types of fractures that patients with osteoporosis experience.^1^ Vertebroplasty is widely used as a treatment for such fractures, offering vertebral stability and pain relief. The procedure involves the injection of bone cement into the fractured vertebral body, reducing motion and stabilizing the segment.

Two influential blinded randomized and controlled studies by Buchbinder et al^2^ and Kallmes et al^3^ have raised questions over the efficacy of vertebroplasty following their findings that there were no significant differences in outcomes between vertebroplasty and placebo groups. However, there is evidence of positive outcomes to the procedure,^4^ and it may be the case that subgroups of patients with particular characteristics benefit more than others.

Finite‐element (FE) models are a clear choice to investigate how different types of variation in the procedure, geometry and material properties change the effectiveness of vertebroplasty from a mechanical perspective. While a number of studies have attempted to model cement augmentation in human vertebrae,^5^, ^6^, ^7^, ^8^, ^9^, ^10^ few have attempted to validate models against experimentally augmented specimens^8^, ^9^, ^10^ and fewer succeeded in producing models that could closely predict experimental findings.^10^ Studies that achieved good agreement in modeling cement augmentation required individual calibration, meaning that the methods have limited application in analyzing larger sets of vertebral models. There is therefore a need to build validated, specimen‐specific models of augmentation in vertebrae to provide robust predictions of how vertebroplasty affects the population. An understanding of the effects of variation in terms of geometric features, material properties, and changes to the vertebroplasty procedure on the mechanical response to augmentation will allow a determination of which vertebrae are best suited to receiving the procedure.

The primary aim of this study was to develop methodologies to accurately model both augmented and nonaugmented human lumbar vertebrae. The details of the human tissue used in this study are described along with the experimental methods for testing, augmenting, and imaging the specimens. A comparison of two‐image to FE modeling methods is then reported. Comparisons are then made between the experimental and FE results and the implications are discussed.

## 2 METHODS

### 2.1 Experimental methods

A total of 14 human lumbar vertebrae were harvested from four fresh frozen cadaveric spines obtained with ethical permission from the Leeds GIFT Research Tissue Project, as detailed in Table 1.

**Table 1:** Details of the lumbar sections used from four cadaveric spines

Spine name	Vertebrae	Sex	Age
Spine 1	L1, L2, L3, L4, L5	F	90
Spine 2	L1	F	94
Spine 3	L1, L2, L3	M	86
Spine 4	L1, L2, L3, L4, L5	M	83

A simulated prophylactic vertebroplasty procedure was undertaken on each vertebra; all specimens were also mechanically tested and imaged before and after the augmentation, as described below.

**Figure 1:**
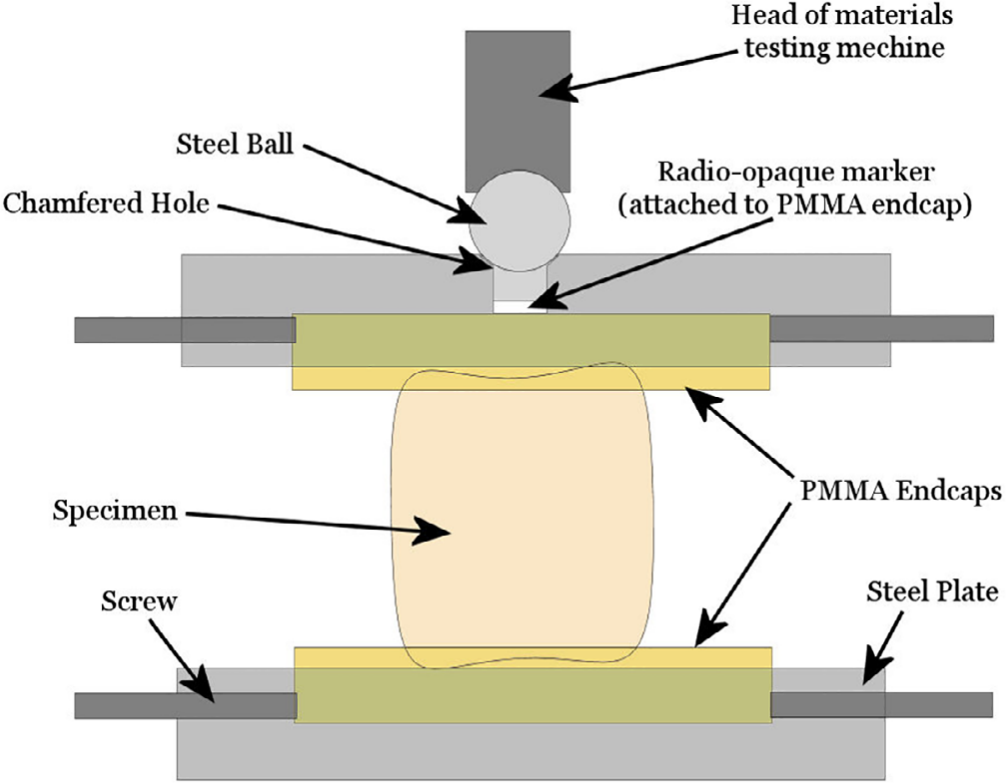
The experimental setup for axial loading the vertebral specimens

The mechanical stiffness of the vertebrae were determined under axial compression using a materials testing machine *(*Instron 3366, 10 kN load‐cell; Instron Ltd, UK). Stiffness was chosen for the measured outcome as the pain reduction following augmentation is thought to originate through a stabilization of the vertebrae.^11^ Each vertebra was first potted in polymethylmethacrylate (PMMA) endcaps, and placed between two steel end plates, the lower of which inhibited lateral motion of the specimen when under load. A ramp load up to 1600 N was then applied at a rate of 1 mm/min to avoid damaging the vertebrae and viscoelastic effects. The load was applied through a steel ball allowing flexion of the top steel plate (Figure 1). The stiffness of the vertebrae was measured by identifying the maximum gradient in the linear region of the load displacement curve, by iteratively calculating the gradient over segments of 20 data points (equivalent to a displacement of 0.034 mm). All specimens were imaged in their PMMA endcaps before and after augmentation using high resolution peripheral quantitative computed tomography (HR‐pQCT; XtremeCT; Scanco Medical AG, Switzerland) with an isotropic voxel size of 82 μm, energy settings of 900 lA, 60 kVp, and 300 ms exposure time. A radio‐opaque marker was embedded in the upper endcap to align with the position of the steel ball (Figure 1), enabling the location of the applied load to be identified on the images.^12^, ^13^

**Figure 2:**
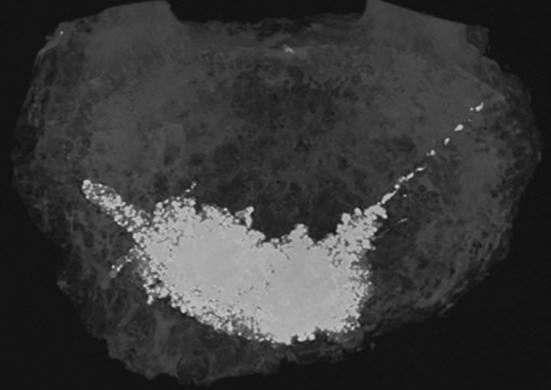
A micro‐computed tomography scan showing the injected volume of cement at the anterior of the vertebral body with the cement track from the exiting needle to the right. For this specimen, cement leaked through the anterior wall, limiting the quantity of cement injected

Augmentation of the vertebrae used an oblique approach, avoiding damage to the dense cortical bone surrounding the pedicles (Figure 2). A side‐opening needle was used, allowing the cement to be directed into the anterior center region of the vertebral body as opposed to directly out of the needle end. The cement used was PMMA, mixed in a powder:liquid weight ratio of 1:1, with barium sulfate in a 1:4 ratio with the powder component, as a radio‐opacifier. The largest possible volume of cement was injected into each vertebra, mimicking the clinical process. The injection was stopped upon observed cement leakage, which generally occurred through one of the vascular channels at either the posterior or anterior portions of the vertebrae. The resulting cement fill volume was measured using segmented micro‐computed tomography (μCT) scans.

### 2.2 Computational methods

#### 2.2.1 Nonaugmented models

**Figure 3:**
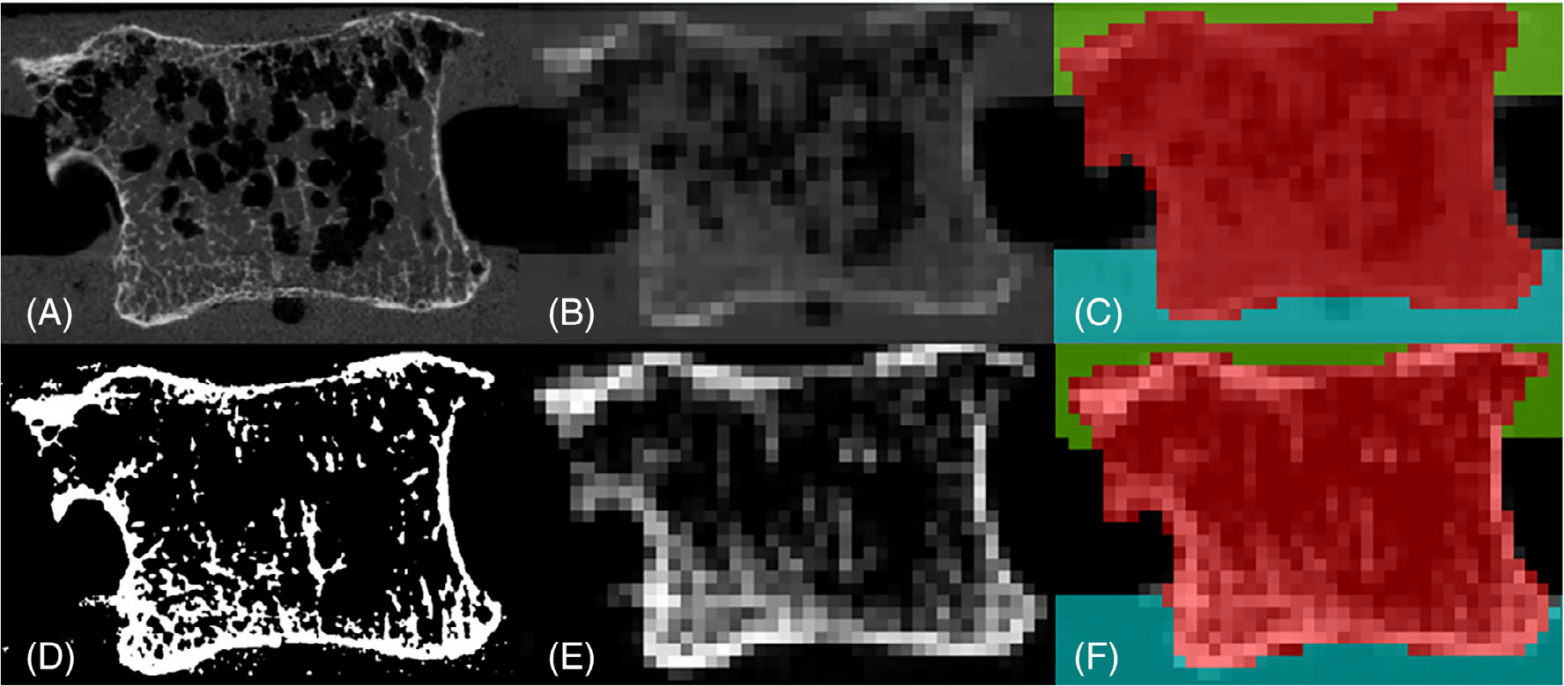
A comparison of the direct grayscale method, A‐C, and the bone volume fraction method, D‐F. A, The original full (82 μm) resolution scan. B, The same image downsampled to 1 mm resolution. C, The resulting segmented scan. D, The segmented bone at 82 μm. E, The segmented image of D following downsampling to 1 mm. F, The resulting image after segmentation

FE models were generated based on μCT image data, converted into 8‐bit TIFF format image stacks using an in‐house script written in MATLAB (Mathworks, USA). Image processing was carried out using Simpleware ScanIP (version 7; Synopsis, USA) and ImageJ (Fiji version 1.51a, https://imagej.net/Citing)^14^ for masking and meshing, and for threshold determination, respectively. Two approaches to generating the models were compared (Figure 3). In the first (“direct grayscale method”), the images were downsampled to 1 mm^3^ and the resulting grayscale of each voxel was used to define the local bone elastic modulus (Figure 3A,B), similar to a method used by Zapata‐Cornelio et al.^15^ In the second (“bone volume fraction” method), an initial threshold was applied to the 82‐μm image stack to segment the trabeculae from the trabecular spaces (Figure 3D). The selected threshold was determined as the mean across all specimens using the *optimize threshold* feature of the BoneJ plugin for ImageJ (version 1.4.1^16^), which optimized the connectivity against the threshold value. The segmented images were then downsampled to 1 mm^3^ such that the resulting grayscale value of each voxel, which was used to define the material properties, was proportional to the regional bone volume fraction (Figure 3D,E) similar to the method reported by Robson Brown et al.^17^ In both cases, the downsampled images were then segmented to separately mask the bone and endcap regions (Figure 3C,F).

FE meshes were generated for the two approaches in ScanIP using a mix of hexahedral elements internally and tetrahedral elements on the mesh surface for smoothing. Meshing was at the model μCT background resolution of 1 mm^3^, given the results of previous studies.^18^

Bone materials were modeled as an isotropic linear elastic material, where the Young's modulus of each element was correlated with the grayscale value of the corresponding voxel using a linear conversion factor. The relationship between the grayscale value and Young's modulus was optimized to provide the best fit between the FE‐predicted stiffness values and the corresponding experimentally derived stiffness values using an optimization toolbox^19^ with the method employed by Zapata‐Cornelio et al.^15^ Initially, the 14 vertebra models were split evenly into a build set and a validation set, with the optimization of the conversion factor carried out on the build set and this factor was then applied to the models in the validation set. Following this validation step, all 14 vertebrae were then used to optimize the conversion factor. The remaining properties used for all of the models are listed in Table 2.

**Table 2:** Material properties used for the vertebral models

Material	Young's modulus (GPa)	Poisson's ratio	Yielding stress
PMMA endcaps^20^	2.45	0.3	—
Augmentation cement	1.7	0.4	—
Augmentation cement interface	0.01	0.4	5 Pa → P.P.

*Note*: Properties for the cement and cement interface were tuned using the described method. Properties for the endcaps originated from the literature.^20^

Abbreviations: PMMA, polymethylmethacrylate; P.P., perfectly plastic behavior.

Constraints were set such that the inferior endcap had an encastre boundary condition matching the steel plate housing in the experimental setup. A rigid platen was tied to the superior endcap with a point load applied through the plate at a location matching the experiment for each specimen. The load was applied using a 1‐mm displacement with rotation allowed and nonaxial translations constrained. The interfaces between the endcaps and bone were defined as frictionless contact, with separation prevented following contact, mimicking the adhesive‐less properties of the PMMA endcaps.

#### 2.2.2 Augmented models

**Figure 4:**
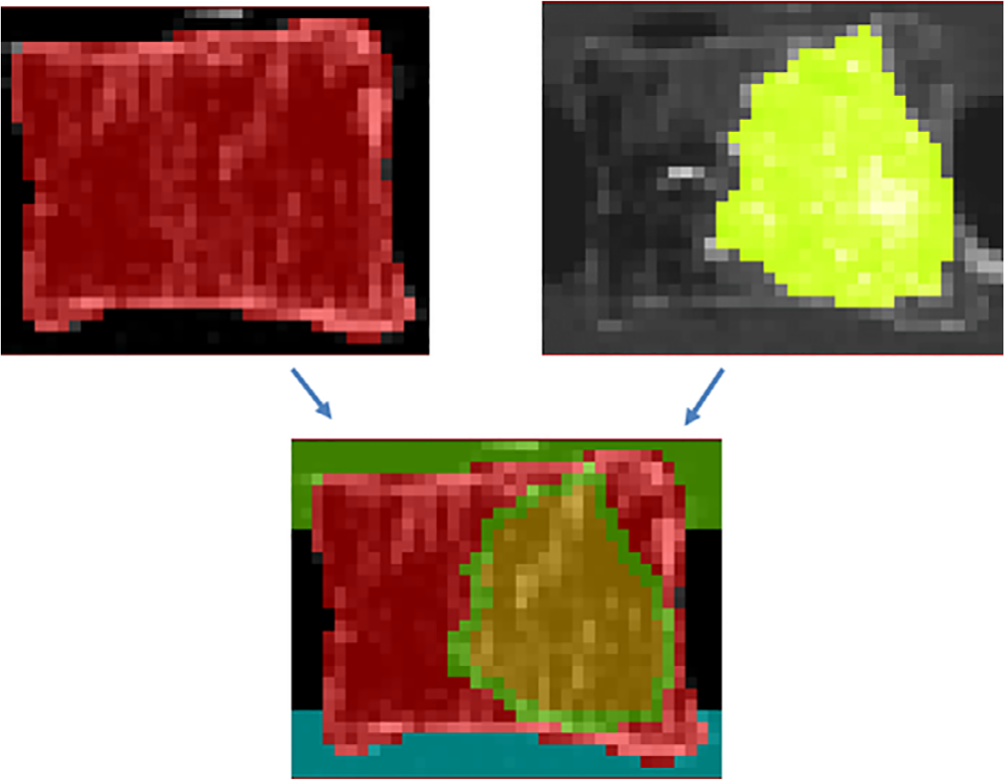
An illustration showing the origin of the masks from the nonaugmented scan (left) and the augmented scan (right)

Two approaches to modeling augmentation were also compared. The first involved a simple segmentation of the four regions in the postaugmentation scans of the vertebrae: the two endcaps, the vertebra, and the region of injected cement. This method effectively uses the direct grayscale method, given that the trabecular bone cannot be segmented from the augmented regions. The second approach utilized image registration, combining scans of pre‐ and post‐augmentation in order to remove artifacts from the barium sulfate and allow use of the presegmented nonaugmented scan data. The cement regions were identified from the augmented scan and superimposed onto the registered nonaugmented scan. The bone material properties were then derived from the nonaugmented initial computed tomography scan. Registration of the images was carried out in 3D Slicer (version 4.10, www.slicer.org 21) using the pre‐ and post‐augmentation scans, using the direct grayscale background version for the preaugmentation scan. The method of registration used three landmarks for each vertebra (the superior of one pedicle, the inferior of the other pedicle, and the inferior anterior of the vertebral body). The selection of these points proved to provide a repeatable registration of the vertebrae, without selecting superfluous landmarks. This registration allowed the two grayscale backgrounds to be used for one model as shown in Figure 4, where the mask and material properties for the vertebrae were derived from the preaugmentation scan and the mask for the cement region came from the augmented scan.

A final variation of the modeling process defined the needle tracks within the vertebral mesh by removing the mask based on the postaugmentation μCT scan. Given that the material properties are based on the nonaugmented image data, the damage created by the needle tracks from the augmentation process would otherwise not be included.

Material properties for the augmented region in all the modeling approaches included a yielding material interface layer between the cement and trabecular bone regions. This was to represent the trabecular level interfaces between the two materials at the continuum scale. The interface layer was created by using the dilate feature within ScanIP, giving two masks, one describing a 1‐mm‐thick layer between the bone and the cement region and the other the cement region itself. Material properties for the region are described in Table 2, which were initially based on the study by Sikora et al,^22^ with subsequent tuning to match the experimental results.

#### 2.2.3 Analysis

All FE analyses were conducted with Abaqus 6.14 (Simulia, Dassault Systemes). Models were run on 10 cores within an High Performance Computing (HPC) cluster, where each model solved within 20 minutes. The agreement between the experimental and computational measures of stiffness was quantified using the concordance correlation coefficient (CCC).^23^

## 3 RESULTS

The dataset associated with this study is openly available from the University of Leeds data repository.^24^

### 3.1 Experimental results

**Figure 5:**
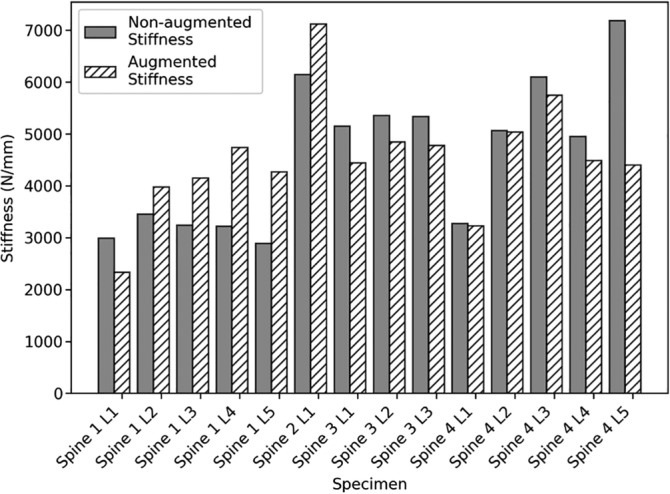
The stiffness variation seen before and after augmentation for the 14 specimens

**Figure 6:**
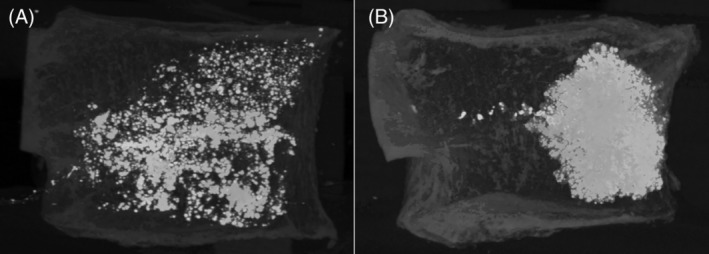
A, Vertebra showing a dispersed volume of cement. B, Concentrated volume of cement

**Figure 7:**
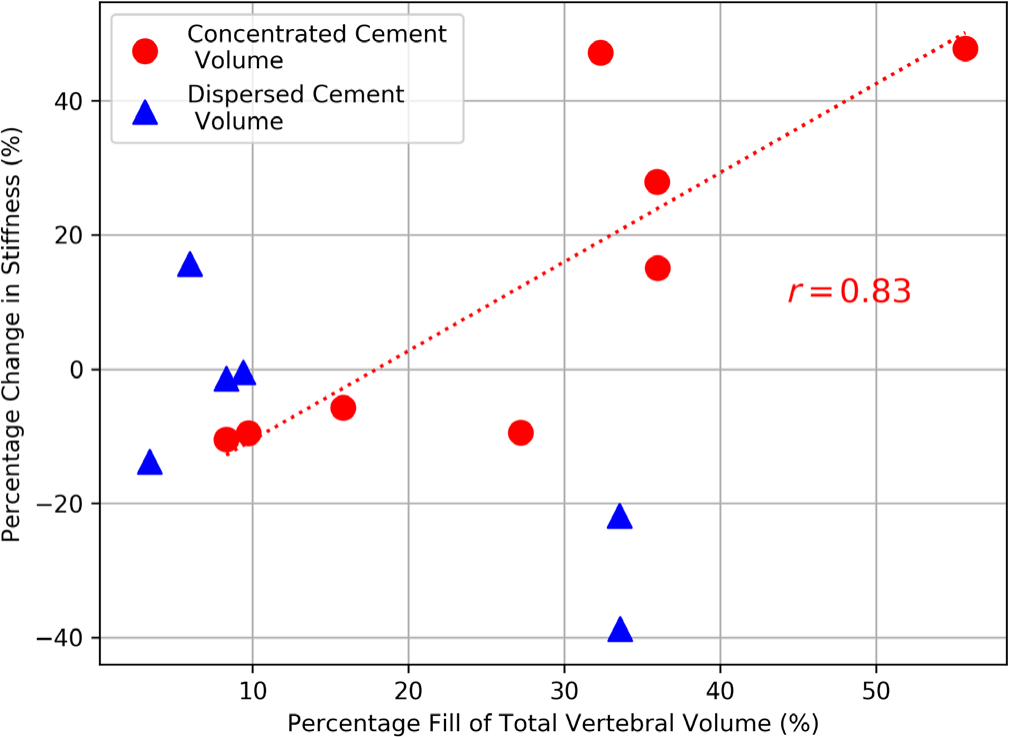
The relationship between the percentage fill of the total vertebral volume and the percentage change in the vertebral stiffness following augmentation. The line and *r* value are for the red points where the cement volume was characterized as concentrated. The remaining blue points indicated the vertebra where the cement volume was characterized as dispersed

A large variation in vertebral stiffness was found following augmentation that ranged from −39% to +48% of the nonaugmented stiffness (Figure 5). The distribution of the cement was found to vary from a concentrated region to being highly dispersed (Figure 6, Table 3). For the vertebrae with a concentrated region of cement, there was a correlation between the change in stiffness following augmentation and the fill volume (Figure 7). In addition, vertebrae with concentrated volumes of cement had a correlation between density of the vertebrae and the quantity of cement injected before cement leakage occurred.

**Table 3:** Bone volume fraction values for the specimens, the achieved augmentation fill volume and the nature of the injected cement volume, dispersed or concentrated

Vertebra	BV/TV	Percentage fill of vertebral volume	Dispersed cement volume
Spine 1 L1	0.174	33	Yes
Spine 1 L2	0.170	35	No
Spine 1 L3	0.137	35	No
Spine 1 L4	0.127	32	No
Spine 1 L5	0.187	55	No
Spine 2 L1	0.391	5	Yes
Spine 3 L1	0.255	3	Yes
Spine 3 L2	0.267	9	No
Spine 3 L3	0.281	8	No
Spine 4 L1	0.257	8	Yes
Spine 4 L2	0.241	9	Yes
Spine 4 L3	0.244	15	No
Spine 4 L4	0.247	27	No
Spine 4 L5	0.249	33	Yes

Abbreviation: BV/TV, Bone Volume Fraction.

### 3.2 Computational results

**Figure 8:**
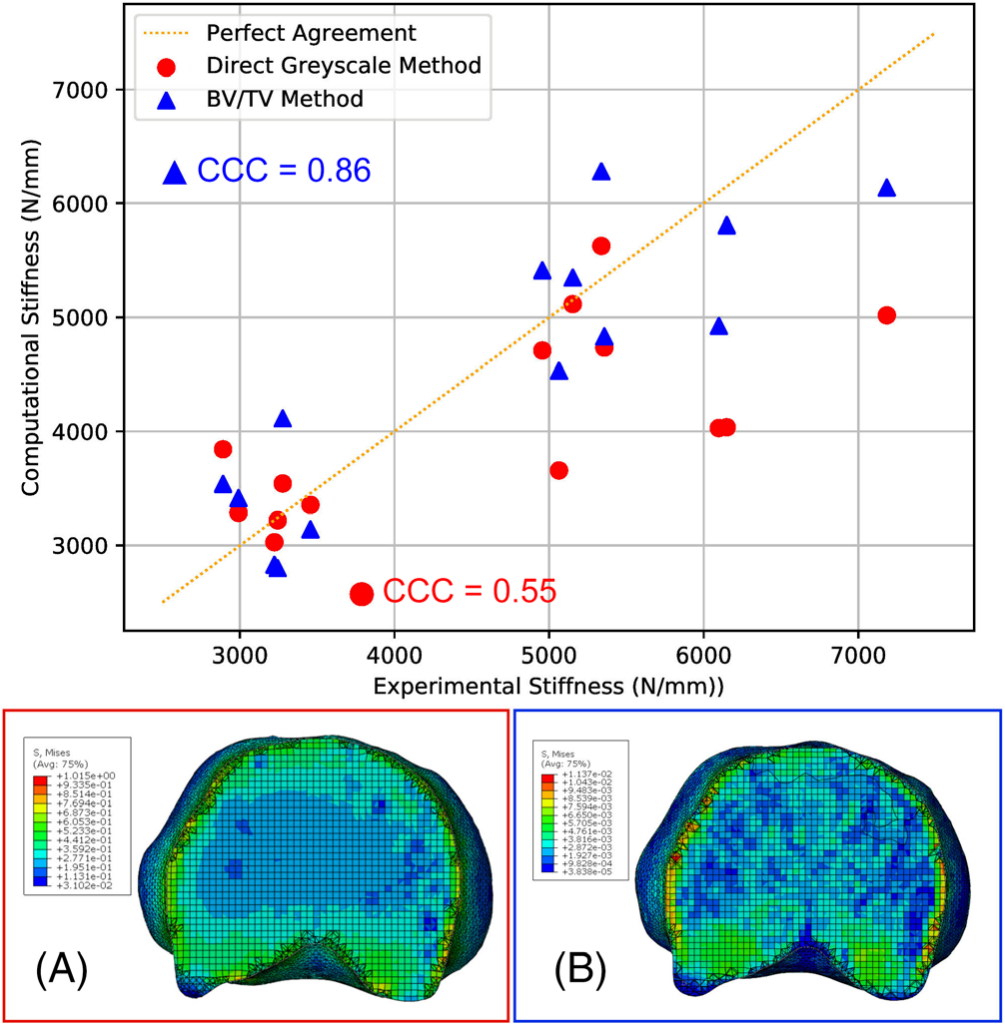
Comparison between the vertebral stiffness predicted from the computational models and the corresponding values derived from the experimental tests for the two different methods of deriving the bone material properties. Red shows the agreement using the direct grayscale method and blue shows the agreement using the bone volume fraction method. The concordance correlation coefficient values for each method are shown along with the line of perfect agreement. A and B, The stress distributions in the mid‐slice of a typical vertebral model (Spine 3 L1). B shows the improved cortical shell load transfer using the bone volume fraction method compared to the direct grayscale method in A

The validation set for the direct grayscale method had a CCC value of 0.53 and the CCC value for the validate set of the bone volume fraction method was 0.83. Utilizing all 14 vertebrae to optimize material property calibration, it was found that there was an improvement in the agreement and reduction in error between the experimental and computational results for the bone volume fraction method (CCC = 0.86, Root Mean Square (RMS) error = 15.3%) compared to the direct grayscale method (CCC = 0.55, RMS error = 20.1%), as shown in Figure 8.

**Figure 9:**
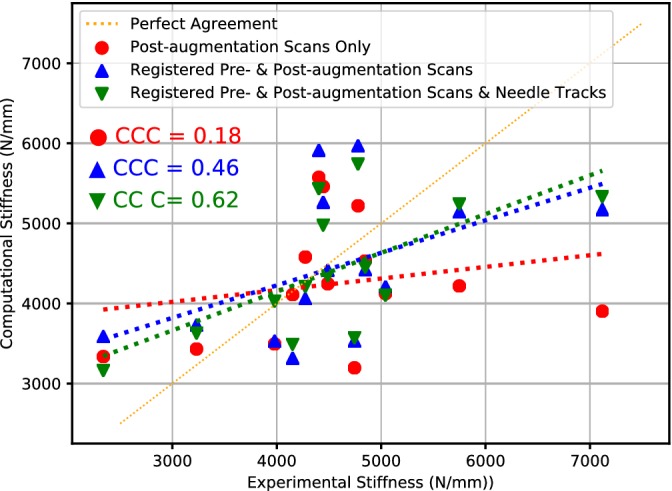
The results of using the initial method (red circles), the registration method (blue triangles), and the defined needle tracks (green triangles), showing the agreement to the perfect *x* = *y* line in orange

In modeling the postaugmentation vertebrae, it was found that the best agreement with the experimental data was achieved using the image registration and the explicitly defined needle tracks (Figure 9). The models developed using this approach achieved a CCC of 0.62 compared to the models that used the registration method but did not include the needle tracks, which achieved a CCC of 0.46, and the initial method utilizing only the postaugmentation scans that had a CCC of 0.18.

**Figure 10:**
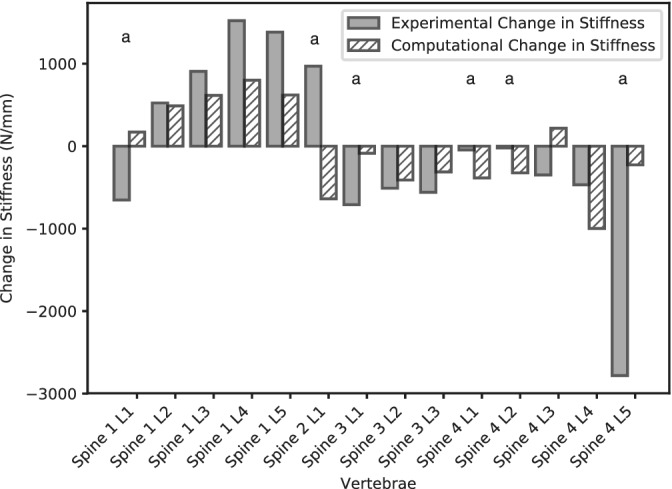
The change in stiffness following augmentation for the experimental specimens and the computational results (using the finite‐element methodology with best concordance correlation coefficient). ^*^The vertebrae received a dispersed volume of cement

The models have the ability to broadly represent the change in stiffness following augmentation (Figure 10). However, a reduction in the model accuracy for those vertebrae that received a dispersed volume of cement can also be observed.

## 4 DISCUSSION

The overall aims of the study were to develop effective methods for modeling augmented vertebrae at the continuum level using image data with experimental data as a comparison. Successful outcomes were achieved for the modeling of nonaugmented and augmented specimens using improved methodologies compared to previous studies and examples found in the literature. Despite this, a number of factors still need to be considered, which are discussed in more detail in the following sections.

### 4.1 Nonaugmented models

In this study, it was found that there was better agreement with experimental results using the bone volume fraction method as compared to the direct grayscale method for generating FE models of vertebrae. The improvement in model agreement using the bone volume fraction method is most likely due to the added definition of the trabecular bone and cortical shell, especially given how important the correct representation of load sharing is for accurate models.^17^ Having greater definition of the cortical shell and trabecular bone alignment also allows a better regional representation of the load transfer through the vertebrae than the more homogenized predictions seen with the direct grayscale method. This improved agreement is much stronger than the agreement found in similar studies that used a comparable methodology to the direct grayscale method^9^, ^15^, ^17^ and comparable to methods that used more complex, specimen‐specific material properties for each model.^10^, ^25^ The conversion between grayscale and Young's modulus was comparable to studies with similar methodologies; Robson Brown et al^17^ found an equivalent conversion factor of 0.0013/GPa (0.0009/GPa in the current study), which gives a similar average bone modulus of 0.33 GPa to that of the current study, 0.25 GPa. Differences between the two are likely from scanner and image processing variances between the studies. An advantage the current study has over the two studies that achieved similar levels of agreement^10^, ^25^ is that it provides a uniform calibration coefficient that, once calibrated over a large set of vertebrae, can be applied to future unseen human lumbar vertebral image data.

The bone volume fraction method also removed any effect caused by the bone marrow, which had leaked in some regions following freeze/thaw cycles. This affected the grayscale‐based models due to regions devoid of bone marrow having a lower grayscale in the trabecular spaces, therefore altering the derived material properties. Due to the initial segmentation and binarization at 82 μm in the bone volume fraction method, the bone marrow was not present in the segmented full resolution or downsampled scans and therefore its presence or otherwise had no effect on the derived material properties of the models.

### 4.2 Augmented models

The results of modeling augmented vertebrae showed a reduction in the agreement when compared to the nonaugmented vertebral models, mirroring that found by Wijayathunga et al.^9^ Modeling augmentation in the vertebral models presents a range of challenges compared to their nonaugmented counterparts, which were addressed here. The challenges included capturing the extent of the augmentation region, modeling the behavior of the regions where the cement to bone ratio is low, and capturing the damage caused by the insertion of the needle.

The difficulty in capturing the extent of the injected cement volume was mainly due to clumping of the barium sulfate particles and separation of the barium sulfate from the other components of the PMMA cement. The clumping of barium sulfate was also found by Sikora.^22^ In the current study, agglomeration was reduced, but not removed, through vigorous mixing of the PMMA monomer and barium sulfate prior to mixing with the PMMA powder. However, others suggested that the separation of the barium sulfate from the cement (calcium phosphate in that study^26^) during the high pressures of the injection was the cause of the subsequent agglomeration. While in the current study this problem was minimized through segmentation of the cement region at higher resolutions before the downsampling, a problem still lies in capturing the intricate interdigitation between the cement and the trabecular bone at a resolution of 1 mm^3^. The use of the registered preaugmentation scans in this study overcame the issues documented by others due to the bright halo of the cement region.^9^, ^22^ This allowed more accurate modeling of the material properties of the vertebral bone and additionally improved accuracy of modeling the interface, given the removal of the false, dense bone surrounding the yielding interface. These improvements are evidenced in the greater level of agreement seen with the experimental data, compared to the use of only the postaugmentation scan data (Figure 9).

The identification of dispersed and concentrated volumes of cement is not limited to this study, Aquarius et al^27^ and Tarsuslugil et al^26^ found similar modes of variation within the achieved volumes of injected cement. As found here, both suggested that the final distribution of cement was governed by the internal bone structure and proposed a relationship with the pressure applied to the cement during injection. The clinically used and commercially available Vebroplast (European Medical Contract Manufacturing, The Netherlands) bone cement used by Aquarius et al also suggests that the effect is not limited to laboratory‐based PMMA mixtures.

The approach used to model the complex cement‐trabecular interactions through the use of yielding interface regions significantly improved the modeling accuracy over simpler descriptions of the augmentation regions. Such regions are difficult to model at the continuum level due to the discontinuity between trabecular regions that are constrained by the cement and those that remain unconstrained. The yielding interface approach follows the results of Sikora,^22^ who showed that the approach improved the accuracy of modeling trabecular bone sections with cement. The improvement in the agreement presented here shows that this approach also works for larger volumes of bone, including whole vertebrae.

The inclusion of the explicitly modeled needle tracks also increased the agreement significantly, with the compromise of interrupting an otherwise automated modeling process due to the difficulty of automatically segmenting the regions.

The models of augmentation were broadly able to represent the change in stiffness following augmentation that was seen in the experiment, with greater variation between the experimentation and computational results for those vertebrae that received a dispersed volume of cement. This is due to the previously discussed difficulty in modeling cement regions that have a low cement to bone ratio. Despite this, the models were able to show the wide range of stiffness outcomes that were seen experimentally, including representing the reduction in stiffness that was present in the densest vertebrae, a result that would not be possible if using a simpler description of the augmented region and its interface. This highlights the remaining challenge in modeling vertebral augmentation at the continuum level: a method of representing the dispersed cement augmentation outcomes.

## 5 CONCLUSION

Overall, the modeling methods presented in this study were found to provide accurate estimates of the stiffness of nonaugmented human lumbar vertebrae. The predictive abilities of models of augmented vertebrae were reduced, although considerable improvements were seen over previous studies that used similar approaches. Importantly, the models were able to represent the large range of outcomes that occur following augmentation, suggesting that the representation is sufficiently robust to examine a range of different augmentation scenarios, including those where the stiffness following augmentation is reduced. This ability will allow future studies to determine which vertebrae are best suited to receiving the procedure from a mechanical standpoint, based on their geometry and material properties.

## CONFLICT OF INTEREST

The authors declare no potential conflict of interest.

## AUTHOR CONTRIBUTIONS

G.A.D., A.C.J., and R.K.W. contributed to the design of the research. G.A.D. completed the implementation of the research and the analysis of the results. G.A.D., A.C.J., and R.K.W. contributed to the writing of the manuscript.
